# A functional spreadable canola and milk proteins oleogels as a healthy system for candy gummies

**DOI:** 10.1038/s41598-022-16809-9

**Published:** 2022-07-23

**Authors:** Heba H. Salama, Ayat F. Hashim

**Affiliations:** 1 Dairy Department, National Research Centre, Dokki, Giza, Egypt; 2 Fats and Oils Department, National Research Centre, Dokki, Giza, Egypt

**Keywords:** Biotechnology, Materials science, Nanoscience and technology

## Abstract

Recently, interest and demand for healthy and useful food products have become a global requirement. Thus, the production of functional foods with high polyunsaturated fatty acids and antioxidants is very challenging. In this study, four functional spreadable oleogels based on canola oil and milk proteins were developed. These spreadable oleogels were used as an innovative model for the preparation of candy gummies. The chemical composition, oxidative stability, and effects of storage conditions were studied. The results showed that the fat content in spreadable oleogels and gummies ranged from 35 to 47 and 2.40–4.15%, respectively. The protein content in spreadable doum and carrot was 7.41%, while it was 6.15% in the spreadable plain and ranged from 10.25 to 12.78% in gummies. The hardness of spreadable oleogels and gummies ranged from 0.3 to 0.9 and 6.22–16.30 N, respectively. Spreadable carrot and spreadable doum had peroxide values greater than 8 meqO_2_/kg after storage, whereas spreadable plain and spreadable canola oleogel had better oxidative stability. The antioxidant activity of spreadable oleogels and gummies ranged from 66.98–46.83% to 51.44–40.37%, respectively. In addition, transmission electron microscopy and polarized light microscopy micrographs showed the presence of a coherent entangled network between oleogels and nutritional polymers. The oil binding capacity of spreadable carrot oleogel had a maximum value of 97.89%, while formed gummies were higher than 99%. This study showed a promising way to make functional spreadable oleogels as a model for food products that are good for health and nutrition.

## Introduction

Fats and oils are widely used in various formulations and diets to improve the nutritional value and properties of food products. Recently, there has been a lot of focus on the health benefits of fats as well as the environmental impacts of their production. Trans and saturated fats produced from oil hydrogenation and fragmentation are used in food products. The consumption of these fats harms human health, including coronary heart disease, endothelial dysfunction, metabolic syndrome, and oxidative stress^1^. Therefore, there is an urgent need to find healthy alternative means to produce solid fats with a low content of saturated fatty acids^2^. One of these promising means is the use of oleogels as a healthy alternative to saturated and trans-fatty acids^1^. Canola oil is considered one of the healthy edible vegetable oils due to its biological effects, cardioprotective substances; reduction in plasma cholesterol levels, and improvement in health in a general^3^. Canola oil has a higher in unsaturated fatty acids than other vegetable oils^4^. Subsequently, it may be more exposed to the oxidation process during storage. The new trend to avoid oil oxidation and maximize its use is the formation of functional oleogels^5^. Oleogels are solidified or semi-solidified materials produced by gelation of oil using several oleogelators (e.g., various waxes, phospholipids, monoglycerides, esters, or alcohols of fatty acids, etc.).

Many studies have used different oleogelators to form the oleogels and protect canola oil. Natural waxes (beeswax, carnauba, and candelilla) and canola oil formed oleogels and also studied their oxidative stability^6^. In addition, candelilla wax and canola oil oleogel improved the texture and reduced the in vitro starch digestibility properties of maize tortillas^7^. Oleogel of canola oil with candelilla wax was prepared and used as a substitute for ghee to produce a high-level alternative to unsaturated fatty acids^8^. Stearic acid has different health advantages when used orally or applied locally^9^*.* It was used as a gelator for the preparation of oleogel using sesame oil and soybean oil^10^. Stable and semi-solid soy lecithin-based oleogels and oleogel emulsions were developed using a combination of soy lecithin and stearic acid as a gelator^11^.

Oleogels act as a vehicle for functional ingredients, such as phytosterols^12^, lecithin^13^, and β-carotene^14^, and trace elements^15^. In addition, protein plays an important and effective role in many vital processes in the human body, building cells and muscles and supplying the body with important amino acids. Besides this importance, the source of protein is also very essential in terms of digestive comfort and absorption quality. Milk proteins are amazing functional proteins that have many health and nutritional values. It is also used as a natural delivery system for many vital compounds to maintain their health benefits and protect them from oxidation and degradation during manufacturing as well as during passage through the digestive system^16^. These proteins are easy to digest and absorb. The average digestibility is about 95%, which is considered one of the highest digestibility for nutritional proteins^17^. In addition, milk proteins have binding sites in their structure to bind different molecules^18^. They had antioxidant activity; they also had many preventive properties to work as assistance in traditional treatments, such as the treatment of cardiovascular diseases, metabolic disorders, bowel health, and chemical protective properties^19^. The added milk proteins in the preparation of spreadable oleogel produced a final product that was homogenous and stable^20–22^.

These oleogels can be used in bakery products and as scalps for breakfast, margarine, chocolate, chocolate products, and some meat products^23,24^. Various oleogel formulations consisting of different techniques are used in several products^25^. Food science uses oleogel widely due to its different applications as a food additive to minimize the level of saturated and trans-fats in food^26,27^. Consequently, the oleogels can be a good opportunity to replace harmful solid fat to formulate healthier food products.

Taking into consideration the above-mentioned facts, the real challenge is using healthy fat alternatives in food production and health supplements. In this study, the fundamental selection of ingredients was based on their natural, nutritional, and healthy properties. The main ingredients were canola oil (source of unsaturated fatty acids), milk proteins (source of nutrition), carrot juice (source of beta-carotene), doum juice, and its extract (source of polyphenols and flavonoids). Thus, spreadable canola oleogels naturally rich in unsaturated fatty acids, protein content, and antioxidants could be used as an innovative model for nutritional healthy gummy formulations. As a result, the goals of this research were to (1) prepare and characterize novel functional spreadable oleogels; (2) use these spreads as a natural nutritional model to develop healthy gummies; (3) investigate the chemical composition and oxidative stability of prepared formulations; and (4) determine the effect of storage on color, oil binding capacity, and pH.

## Materials and methods

### Material

Sorbitol was received from BDH Chemicals Ltd, Poole, United Kingdom. Stearic acid was purchased from Spectrum Chemical Mfg. Corp. Gardena, United States. Starch was obtained from S.D. fine chemical, Boisar, India. Casein was received from VWR International Ltd, Poole, United Kingdom. Whey protein concentrate (WPC) was purchased from Arla Foods Ingredients Videbaek. Denmark. The maltodextrin was obtained from Loba Chemie Pvt Ltd., Mumbai, India. Citric acid was gotten from ElNasr Pharmaceutical Chemicals Co., Egypt. Canola oil, doum powder, carrot; gelatin, sugar cane, caramel syrup, and chocolate syrup were purchased from the local market in Giza, Egypt.

### Preparation of an aqueous extraction

#### Doum juice and extract

The crushed doum fruit was boiled in water at a ratio of 1:5 (w/v) for 10 min. The extract was drained through one layer of cheesecloth and pressed to obtain the free-running extract. The produced extract was filtered through a piece of cotton to remove fine particles (doum juice). The same procedures were followed to prepare doum extract by rotary evaporation (doum extract).

#### Carrot juice extraction

The carrot juice extraction was obtained using a mixer grinder. The extracted juice was then filtered and pasteurized at 80 °C for 10 min^28^.

### Preparation of healthy and nutritional spreadable oleogels and candy gummies

#### Preparation of functional spreadable oleogels (O, P, D, and C)

**Figure 1:**
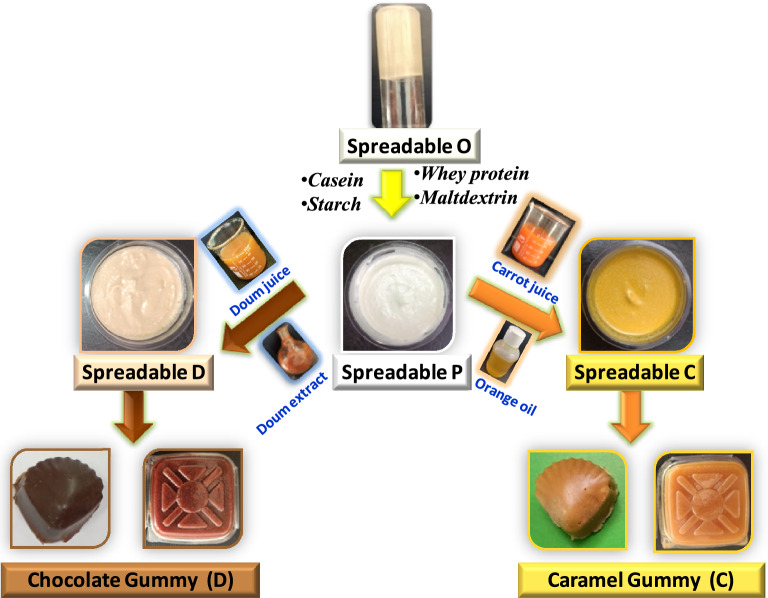
Schematic diagram for preparation of functional spreadable (O, P, D, and C) oleogels and their application in chocolate and caramel gummies.

Figure 1 demonstrates the preparation of functional spreadable oleogels based on canola oil. Canola oil oleogel was prepared using stearic acid as the organogelator. Stearic acid (10%) was dissolved in canola oil in a beaker (100 ml) and heated at 70 °C with stirring until the gelator was completely dissolved in the oil. The hot clear mixture was cooled down to room temperature and then stored at 4 °C in a refrigerator for further analysis (spreadable O). 10 g of casein powder was dispersed in 100 mL of Milli-Q water and stirred for 2 h at room temperature. Whey protein solution (10% w/v) was added dropwise to the previously prepared suspension while being stirred on a stirring plate. Then, a 1:1 starch and maltodextrin were added to the casein-whey protein mixture and stirred for 5 min. A high-speed homogenizer (POLYTRON^®^ PT10-35GT, Kinematica AG, Switzerland) was used to mix the obtained mixture with previously prepared spreadable O for 5 min at 11,000 rpm. After homogenization, the spreadable plain oleogel was produced (spreadable P). The same procedures were followed for the preparation of functional spreadable oleogel, either spreadable doum oleogel (spreadable D) or spreadable carrot oleogel (spreadable C). In the case of spreadable D, the doum juice and its extract were used instead of water, but for spreadable C, carrot juice and orange oil (4% w/v) were used. All the different prepared spreadable (P, C, and D) oleogels were heat-treated at 85 °C for 5 min, then cooled and stored at 4 °C in a refrigerator until further investigation.

#### Formulations for healthy nutritional candy gummies

The healthy nutritional candy gummy formulations were produced based on the previously prepared functional spreadable oleogels. The formulations for different healthy nutritional gummies were listed in Table 1, and the procedures are outlined in Fig. 1. The sugar syrup was prepared by mixing chocolate or caramel syrup; sugar, sorbitol, and citric acid with water. The syrup mixture was heated to 118 °C till the solid content reached 88–90 Brix, measured through a refractometer (Atago Co., Ltd., Japan). Granulated gelatin was hydrated for 30 min in cold water and melted for 1 h in a water bath at 60 °C. The melted gelatin was added to the previous syrup mixture at 100 °C, stirring for 3 min to mix well. The functional spreadable oleogel (P or D or C) was then added to the sugar-gelatin mixture and stirred for 10 min to disperse the oleogel. The different final prepared gummy formulations were deposited separately into a silicone mold at 60 °C. The developed gummies were kept in the silicone mold at room temperature in the dark for 24 h before being removed. Two batches of chocolate gummies were made: gummies based on spreadable P (gummy P_cho_); and gummies based on spreadable D (gummy D). Two other batches of caramel gummies were prepared: gummies based on spreadable P (gummy P_car_) and gummies based on spreadable C (gummy C).

**Table 1:** Functional spreadable oleogels and healthy nutritional gummy formulations.

Functional spreadable oleogels				
Ingredients %	Spreadable P	Spreadable D	Spreadable C	
Spreadable O	50	50	50	
Casein	10	10	10	
Whey protein	10	10	10	
Maltodextrin	3	3	3	
Starch	3	3	3	
Water	24	–	–	
Doum juice	–	20	–	
Doum extract	–	4	–	
Carrot juice	–	–	20	
Orange oil	–	–	4	

Ingredients %	Healthy nutritional gummies formulations			
	Chocolate (%)		Caramel (%)	
	P	D	P	C
Spreadable P	10	–	10	–
Spreadable D	–	10	–	–
Spreadable C	–	–	–	10
Chocolate syrup	38.62	38.62	–	–
Caramel syrup	–	–	38.62	38.62
Sorbitol	6.10	6.10	6.10	6.10
Sugar	29.47	29.47	29.47	29.47
Water	3.43	3.43	3.43	3.43
Gelatin	6.84	6.84	6.84	6.84
Water	3.69	3.69	3.69	3.69
Citric acid	1.85	1.85	1.85	1.85

### Characterization

#### Fatty acid composition and physicochemical characteristics of canola oil

The fatty acid composition of canola oil was determined using a Hewlett Packard HP 6890 gas chromatograph, a flame ionization detector (FID), and a capillary column (30 m × 530 μm, 1.0 μm thickness). A carrier gas, nitrogen, was set at a flow rate of 15 mL/min while the temperature of the injector and detector was set at 280 °C. The temperature of the column was maintained at 240 °C. The peaks were identified as compared to chromatograms of standard fatty acid methyl esters (Sigma, USA). The physicochemical characteristics of canola oil (refractive index, peroxide, acid, iodine, and saponification value) were determined according to^29^.

#### Chemical composition analysis

The fat, protein, ash, total solids, total protein content, and pH were determined according to^30^.

#### Measurement of viscosity

All the viscosity studies were measured at room temperature (25 ± 1 °C) using an Ametek Brookfield digital viscometer (Middleboro, MA 02346, USA). The samples were subjected to a cyclic shear rate in the range of 3 s^−1^ to 100 s^−1^ for spreadable (P, D, and C) and S^−6^ for spreadable O for an upward curve^31^. The viscosity was expressed as centipoise (cP s).

#### Texture assessment

Texture profile analysis (TPA) was performed on samples using a double compression tester (Multitest 1d Memes in, Food Technology Corporation, Slinfold, W. Sussex, UK). All of the determined parameters (Hardness (N), Cohesiveness, Adhesiveness (N.s), Springiness (mm), Gumminess (N), and Chewiness (mJ) were determined using the International Dairy Federation's (IDF, 1991) definition. The samples were prepared and measured as previously described by^32^.

#### Oxidative stability

##### Peroxide value

The functional spreadable oleogels (O, P, D, and C) were stored in 20 ml glass vials at 4 °C for 60 days. 6 g of spreadable oleogels were placed into a 100 ml beaker, suspended in 60 ml of n-hexane, and shaken until complete dissolution. The mixture was filtered and the solvent was evaporated under reduced pressure until a constant weight was obtained. The peroxide value was determined iodometrically according to^29^ and was evaluated every 15 days.

##### Radical scavenging activity of DPPH

The antioxidant activity assay using the stable 2,2′-diphenyl-1-picrylhydrazyl free radical (DPPH^·^) was determined as done by^33,34^ with some modifications. Spreadable oleogels or gummies (3 g) were stirred in 100 ml of methanol for 1 h, and then filtered using Whatman no. 1 paper. The residue was re-extracted with another 100 ml of methanol for 15 min. Then, 5 μl of the filtrate was added to 995 μl pure methanol and 2 ml of freshly prepared 0.13 mM DPPH solution in methanol. The sample solution was vigorously shaken on a vortex for the 30 s and then immediately placed in a UV/VIS spectrophotometer (T80 UV/VIS Spectrometer, PG Instruments Ltd., UK). The absorbance was measured at 516 nm after exactly 30 min against pure methanol. A blank was made as above by replacing the test sample with 5 μl of methanol. The percentage of radical scavenging activity (RSA %) was calculated using the equation below:

$$RSA (\%)=\frac{{Ab}_{B}-{Ab}_{S}}{{Ab}_{B}}*100$$

where Ab_B_ and Ab_S_ are the absorbance values of the blank and sample, respectively. The measurements were carried out in duplicates.

#### The effect of storage conditions

The effect of storage conditions at room temperature (30 °C) and refrigerator (4 °C) on chemical composition (“Chemical composition analysis”), color, pH, and oil binding capacity (OBC) for nutritional gummy formulations was studied. These formulations were stored in capped glass vials (22 ml) and kept at 30 °C in the dark and 4 °C for 12 weeks. Samples were taken at 0 and 12 weeks for visual observation. Spreadable oleogel samples were kept at 4 °C for 60 days.

##### Color stability

The color of the prepared spreadable oleogel and gummies was determined with a Minolta CR-10 Plus colorimeter (Konica Minolta, Tokyo, Japan). A 10 mm thick gummy slice was placed in a transparent round container against a white background. Reflectance was measured on the gummy surface. The result was expressed as CIELAB values L* for lightness, + a* for redness or greenness (− a*), and + b* for yellowness or blueness (− b*). The hue angle (h), chroma (C*), and total color differences ΔE were calculated according to the following equations^35^ where ΔL*, Δa*, and Δb* are the luminosity, redness, and yellowness intensity difference from the initial samples. All measurements were performed in triplicates.

$$\Delta {E}^{*}={(\Delta {L}^{*2}+\Delta {a}^{*2}+\Delta {b}^{*2})}^{1/2}$$

##### pH measurement

pH values were measured using a digital laboratory Jenway 3510 pH meter, UK. Bibby Scientific LTD. Stone, Staffordshire, ST15 OSA.

##### Oil binding capacity (OBC)

The OBC method was adapted from^36^. The melted spreadable oleogels/gummies (1 ml) were placed into a previously weighed Eppendorf tube (weight a) and put in the refrigerator for 1 h. Then the tube was weighed again (weight b) and centrifuged at 9160*g* for 15 min at ambient temperature. After centrifugation, the tubes were turned over onto a filter paper for drainage of released oil and were weighed (weight c) again. The OBC was calculated by the following equation.

$$Released\;oil\left(\%\right)=[(b-a)-(c-a)]/(b-a)*100$$

$$OBC\left(\%\right)=100-Released\;oil\left(\%\right)$$

#### FTIR analysis and morphological observation

##### FTIR analysis

FTIR spectra of the formed spreadable oleogel and gummies were acquired on FTIR Bruker Vertex 80v (National Research Centre, Egypt) with a resolution in the range of 4000–400 cm^−1^.

##### Polarized light microscopy (PLM)

The microstructures of prepared spreadable oleogel and gummies were observed using a polarizing light microscope (Model BX51-P, Olympus, Japan). The spreadable oleogels (20 μl) were deposited on a slide and covered immediately with a cover slip. For the gummy samples, the samples were heated at 90 °C until completely melted. 20 μl of the hot mixture was dropped on a pre-heated glass slide and gently covered with a pre-heated cover slip.

##### Transmission electron microscopy (TEM)

Samples were placed on a copper grid, then coated with carbon (carrier powder), and left to dry at room temperature. Transmission electron microscopy, (TEM, JEOL, JEM, 1230 Japan) working at 100 kV.

#### Organoleptic evaluation

The organoleptic test for spreadable oleogels and gummies was determined by the twenty members of the Dairy Department and Fats and Oils Department at the National Research Centre, Egypt. Samples were tested in terms of appearance, flavor, texture, and color. The organoleptic scores were followed as described by^37^.

#### Proximate costs

The proximate cost of different products targeted in this study was calculated as described by^38^ by considering all the costs of raw materials and processing charges at the laboratory level.

### Statistical analysis

A statistical analysis of the data was performed using Statistica 6.0 software (Stat Soft Inc., Tulsa, Oklahoma, USA). Mean values were compared using the Duncan multiple range test and judged at the P ≤ 0.05 level.

## Results and discussion

### Fatty acid composition and physicochemical characteristics of canola oil

The fatty acid composition and physicochemical characteristics of canola oil were presented in Table 2. The data showed that 11 fatty acids were identified, which represented 99.96 g × 100 g^−1^ of total fatty acids. The oleic acid and linoleic acid represent 60.64 g × 100 g^−1^ and 23.75 g × 100 g^−1^ of total fatty acids, respectively. These results showed that the canola oil has a high unsaturated fatty acid content of about 93.65 g × 100 g^−1^ of the total content and a very good ratio of omega-6/omega-3 (2/1). The results on fatty acid composition were similar to those obtained by^39^. This index confirmed the health characteristics of canola oil and distinguished it as a vegetable oil with a high-quality fatty acid profile^4^. The data also showed that canola oil has a very low content of saturated fatty acids, so it was superior to other vegetable oils (e.g., flaxseed, walnut, etc.)^40,41^. The high content of monounsaturated fatty acids was similar to that of olive oil and helps the right functioning of various physiological systems in the human body. The amount of omega-3 fatty acids (C18:3), was increased in comparison to other vegetable oils (olive, corn, sunflower, etc.)^42^. Canola oil showed the best physicochemical properties: refractive index (1.466 ± 0.001), peroxide value (3.5 ± 0.03), free fatty acids (0.15 ± 0.002), iodine value (102.52 ± 0.03), saponification value (187.05 ± 0.07)^43^.

**Table 2:** Fatty acid composition and physicochemical characteristics of canola oil.

Fatty acid composition	
Fatty acids	Content (%)
Myristic acid (C14:0)	0.04
Palmitic acid (16:0)	3.60
Stearic acid (18:0)	1.45
Palmitoleic acid (16:1)	0.18
Oleic acid (18:1)	60.64
Linoleic acid (18:2)	23.75
Alpha Linolenic acid (18:3)	8.24
Arachidic acid (20:0)	0.74
Erucic acid (22:1)	0.84
Others	0.48
Saturated fatty acids (SFA)	5.83
Monounsaturated fatty acids (MUFA)	61.66
Polyunsaturated fatty acids (PUFA)	31.99
PUFA/SFA	5.48
MUFA/PUFA	1.92
USFA/SFA	16.06
C18:2/C18:3	2.88

Parameters	Values mean
Refractive index at 25 ± 1 °C	1.466 ± 0.001
Peroxide value (meqO_2_/kg)	3.5 ± 0.03
Free fatty acids (%)	0.15 ± 0.002
Iodine value (Hanus) (gI_2_/100 g)	102.52 ± 0.03
Saponification value (mg KOH per g)	187.05 ± 0.07
Relative density (g/cm^3^)	0.918 ± 0.002

### A functional spreadable canola oleogels and candy gummies

The development of oleogels with various compositions enhances their possibility of use in a variety of industries such as pharmaceutical, cosmetic, and food products^44^. In this study, canola oleogel was produced using canola oil and stearic acid as a gelator. At a higher temperature, the stearic acid solution was transparent in canola oil. The spreadable O was formed by cooling the solution at room temperature and confirmed using the inverted tube method^45^. Three functional spreadable (P, D, and C) oleogels were prepared based on the spreadable O oleogel using nutritional polymers (casein, whey protein, maltodextrin, and starch). Spreadable P was used as a plain and showed white-colored gel. Spreadable D formed a brownish-colored gel due to the doum fruit that is used as a source of polyphenols and flavonoids. Spreadable P had an orange-colored gel when carrot juice was used as a source of beta-carotene. Also, four types of healthy nutritional gummies were formulated based on three functional spreadable (P, D, and C) oleogels: Gummy P_cho_, Gummy D, and Gummy P_car_, Gummy C. The chemical composition, oxidative stability, antioxidant activity, storage effect conditions, morphological features, and organoleptic tests were studied.

#### Chemical composition

**Figure 2:**
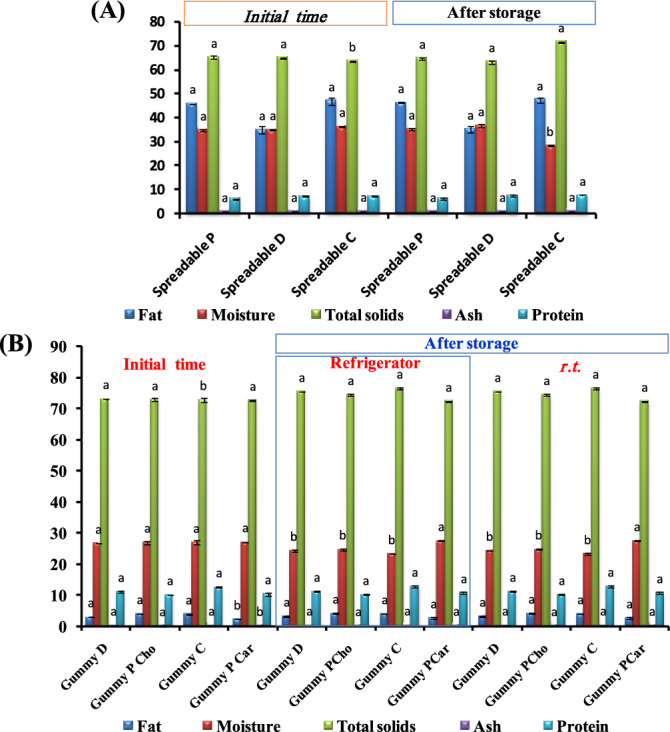
Chemical analysis of (**A**) functional spreadable oleogel and (**B**) healthy nutritional gummy formulations. Columns with similar letters are not statistically different according to DMRT (p ≤ 0.05). Statistical analysis was performed to determine the significance at the initial time and after storage.

Estimating the chemical composition of the food product is an important test as it gives the consumer an impression of the content of the nutrients that they eat, such as protein, fat, and other nutrients. Figure 2 refers to the chemical analysis of different functional spreadable oleogels and gummies. The fat content in spreadable oleogels ranged from 35 to 47%, while it was from 2.40 to 4.15% in gummies. The moisture content of the spreadable oleogels is higher (34.8–36.37) than that of the gummies (26.75–27.27). The higher the total solid content, the lower the moisture content. After storage, the moisture slightly decreased while the solids increased in gummies. The results of gummy moisture agreed with those obtained by^37^, who prepared gummy candy with carrot and pineapple juice. One of the most important ingredients in these products, whether spreadable or gummies, is their protein content, and the source of that protein is milk proteins. The protein content is higher in spreadable D and C (7.41%) than in spreadable P (6.15%). The content of protein increased in gummies in the range of 10.25–12.78%. The highest protein content was also noted with gummy C and gummy D, which contain carrot juice and doum juice and its extract. This increment in protein content may be due to the protein content in carrot and doum. After the storage period, the protein content slightly increased to a range of 10.29–12.97%. This may also be due to a slight increase in the total solids that increased due to evaporation and slightly losing of water during storage.

The ash contents are also presented in Fig. 2. The ash content was high in the oleogel spreadable samples compared with gummies. The highest ash content was found with spreadable C and gummy C, but the lowest content was with spreadable P and the gummies that were made by it (gummy P_Cho_ and gummy P_Car_)_._ During storage, the ash content slightly increased. The higher ash content in the samples that contain carrot juice and doum may be due to the ash content in them compared with those that do not contain them. The results of ash also agreed with^37^. These products, whether spreadable or gummy are considered a good source of functional, healthy, and nutritional protein (milk protein (casein & whey)). They are also a rich source of omega-3 and 6 healthy fats (canola oil).

#### Viscosity measurements

**Figure 3:**
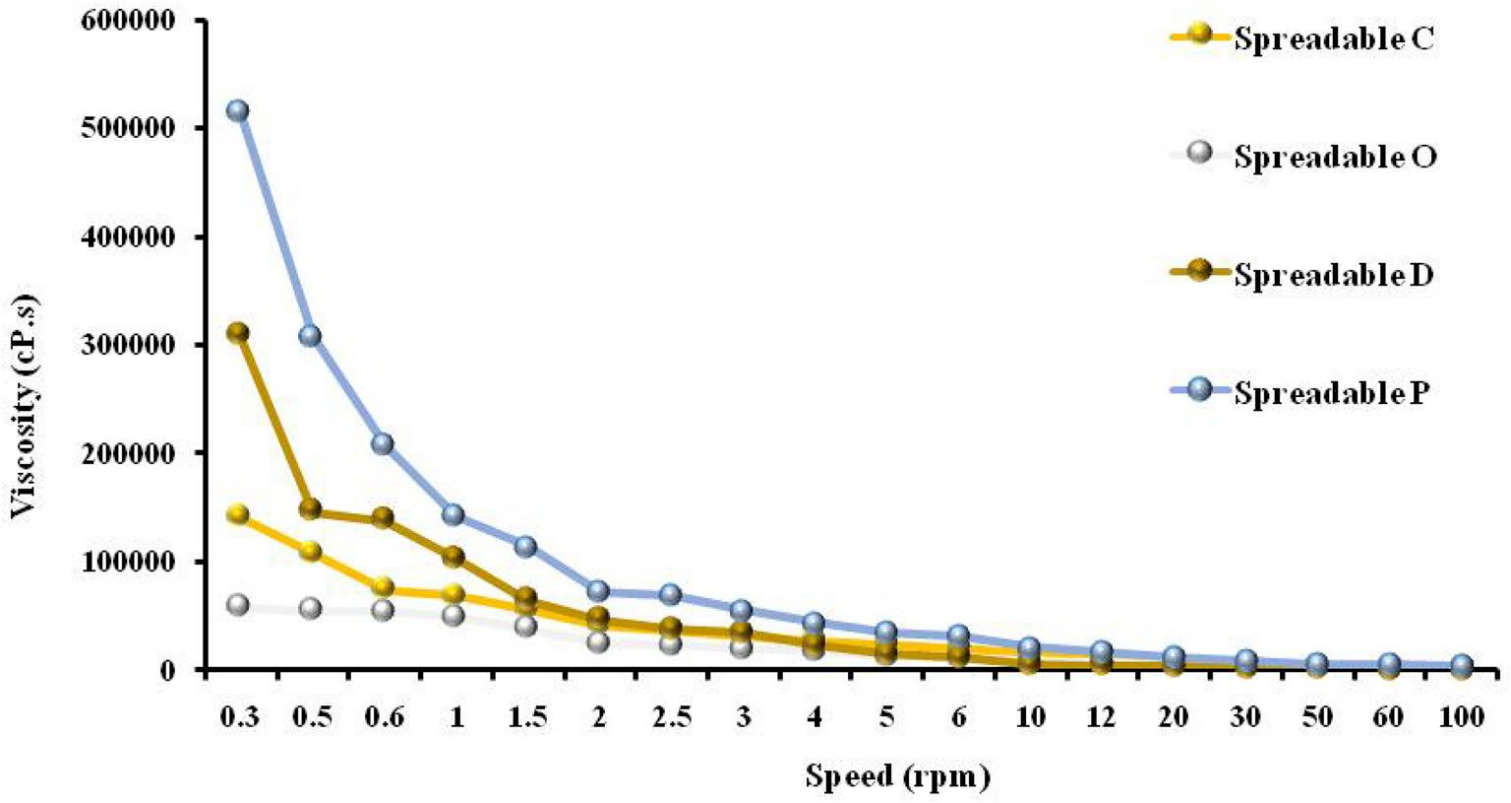
Viscosity of functional spreadable (O, P, D, and C) oleogels.

Viscosity testing is a tool used in the production process of every type of food and beverage. It is a very important test that offers repeatable and reliable results, ensuring that the quality of the product is not only achieved but maintained from batch to batch. From Fig. 3, the viscosity of the spreadable P was the highest one among all tested spreadable samples, followed by the spreadable D, then spreadable C, and the lowest one was spreadable O. The increase in viscosity at the spreadable P may be due to the strong network and interaction between milk proteins (casein and whey protein) with the other polymers (maltodextrin and starch). In addition, the bonds between the canola oil and stearic acid (as gelators) were formed to prepare the oleogels. The increase in viscosity at the spreadable D may be due to the use of doum juice and its extract affecting the acidity and total solids of the mixture and also dispersion and spacing between molecules^31,46^. Carrot juice and orange oil affect the acidity of the spreadable C due to the addition of orange oil and citric acid, leading to a decrease in the flow of the mixture, which affected the viscosity by decreasing.

The combination of different polymers (casein, whey protein concentrates, and maltodextrin) that are added to spreadable oleogels plays an important role in improving the viscosity of the different spreadable oleogels. This may be made by strong networks that bind the oil after its conversion using stearic acid (oleogelator). Casein and whey protein also have binding sites in their structure to bind different molecules^18,47^. Maltodextrin is considered a promising polymer and is commonly used for gelation of the spreadable oleogel. It has many properties such as thickening, stabilizing, and gelling properties, as well as hydrocolloids^48^.

Besides their health and nutritional effects, milk proteins can also play an important role as a gelator. Milk proteins have a special nature; they are hydrophilic to bind water and therefore can crystallize and form a gel, as occurs in different dairy products^49^. Also, milk proteins have hydrophobic properties^50,51^. Whey proteins exhibit different functionalities like emulsification and gelling according to the physicochemical treatment (pH, pressure, temperature) to which they are subjected^52,53^. These functional properties of whey protein are closely associated with the composition and amino acid sequence of these proteins, which allow a specific role in food to be satisfied, for example, providing nutritional value or increasing solubility, gelation, or emulsification in different matrices^54^.

#### Texture measurements

The texture profiles of different functional spreadable and gummies are presented in Table 3. Hardens was defined as the force that breaks or ruptures the samples. Hardens of spreadable oleogels ranged from 0.3 to 0.9 N, while the gummies were from 6.22 to 16.30 N. In the case of spreadable, the highest hardness was noted with spreadable P, while it was high with gummy P_Car_ in the case of gummy, and the lowest was the spreadable O. The gummies are described by their chewy texture as a gelling function of gelatin and other materials like starch and maltodextrin^55^. The gumminess and chewing results are in the same line for both spreadable and gummies. The highest gumminess and chewing were obtained with spreadable P and gummy P_Car_. The high total solids in the gummies reflect on the texture like hardness, gumminess, and chewing. In addition, the speed of forming a strong network to reserve different ingredients and nutrients within this gelatinous network^56,57^. Chewing increases as the content of the solids in the product increases, and this is taken as a sign of water activity^58^.

**Table 3:** Texture profile analysis of functional spreadable oleogel and healthy nutritional gummy formulations.

	Hardness (N)	Cohesiveness (B/A area)	Springiness (mm)	Gumminess (N)	Chewiness (N/m)
Spreadable O	0.3 ± 0.1	0.773 ± 0.001	1.367 ± 0.005	0.232 ± 0.001	0.317 ± 0.009
Spreadable P	0.9 ± 0.15	0.809 ± 0.001	0.883 ± 0.006	0.728 ± 0.003	0.643 ± 0.001
Spreadable D	0.4 ± 0.1	0.866 ± 0.001	0.927 ± 001	0.346 ± 0.1	0.321 ± 0.005
Spreadable C	0.3 ± 0.1	0.613 ± 0.003	0.611 ± 002	0.184 ± 0.2	0.0112 ± 0.003
Gummy D	6.23 ± 0.2	0.804 ± 0.002	0.929 ± 001	5.0008 ± 0.15	4.0652 ± 0.001
Gummy P_Cho_	6.22 ± 0.01	0.797 ± 0.001	0.910 ± 0.001	4.957 ± 005	4.511 ± 0.001
Gummy C	11.29 ± 0.01	0.758 ± 0.001	0.899 ± 005	8.557 ± 005	7.693 ± 0.005
Gummy P_Car_	16.30 ± 0.1	0.791 ± 002	0.852 ± 00.1	12.893 ± 0.1	10.984 ± 0.002

The concentration of gelatin and pH had a noticeable effect on the structure of gels, and the addition of milk protein concentrate (MPC) and skim milk powder (SMP) changed the structure of gelatin. A structure similar to the pure gelatin gel was observed after the addition of whey protein isolates (WPI). The addition of SMP and MPC increased the rheological properties of gelatin gels, while the addition of WPI had a negative impact on them. The hardness of gelatin gels was improved by adding milk powder and gelatin at a concentration of 5%. The addition of milk proteins with a high gelatin concentration led to the loss of cracking gels^59^.

The presence of polymers, mainly milk proteins (casein and whey), in the basic composition of the gummy candy has the main effect on the texture and composition of the gel. Gelatin is therefore mainly used in the manufacture of this type of gelatin candy to achieve the hardness and appearance preferred by consumers^60^. The presence of proteins as well, especially milk proteins, works to provide a strong network in that type of this dessert, which supports and earns it a more solid form and consistency in the presence of other ingredients^60^. The rheological properties have a significant and clear effect on the flavor and acceptance of the product during the immersion of chewing and swallowing. These properties also represent the sensory appearance of the product's textures and the way that texture interacts during application^61^.

Cohesiveness and springiness were also presented in Table 3. Spreadable P and D had the highest values, while in gummies the highest value was noted with gummy D. When springiness is high, it requires more mastication energy in the mouth. High springiness resulted when the gel structure was broken into a few large pieces during the first compression texture profile analysis (TPA), whereas low springiness resulted from the gel breaking into many small pieces^58^. The texture of the different spreadable oleogels was reflected in the results of the viscosity. So, it is suitable for use as fillings for baked products and biscuits and as a surface layer for decorating cakes.

#### Oxidative stability

##### Peroxide value

**Figure 4:**
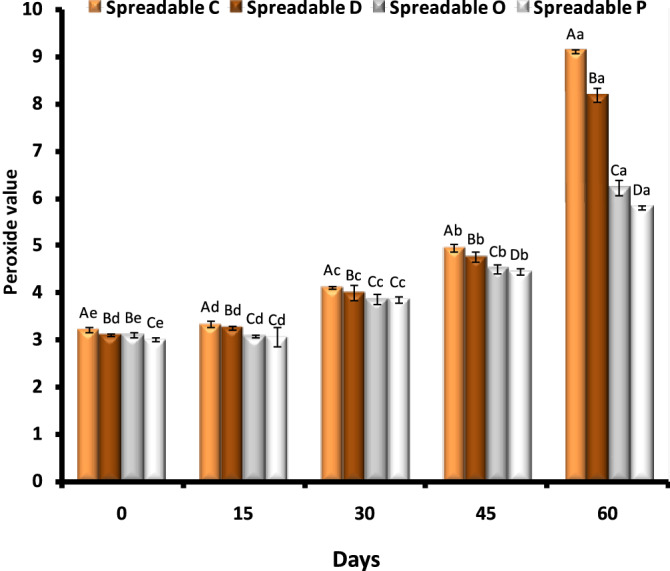
Oxidative stability of the oleogel samples (O, P, D, and C) as evaluated by peroxide value (PV) measurement. Bars represent the standard error of the mean values. Columns with similar letters are not statistically different according to DMRT (p ≤ 0.05). Capital letters refer to the same time, while small letters refer to the same sample at different times.

The oxidative stability of the oleogel samples (O, P, D, and C) was monitored during 60 days of storage at refrigerator temperature (4 °C) by following PV measurements (Fig. 4). The peroxide values of all the samples tended to gradually increase over storage time. These findings are in line with the results of the study by^6^. Among all the stored oleogel samples, the highest PV was measured in spreadable C, followed by spreadable D, while spreadable P and O showed better oxidative stability. Canola oil has more unsaturated fatty acids than other vegetable oils^4^. So, it may be more susceptible to the oxidation process during storage. The new trend to avoid oil oxidation and maximize its use is the formation of functional oleogels. The oleogel technology can be used as a method to prevent oil oxidation by immobilizing oil^62^. Casein is an excellent candidate to prepare oil-in-water emulsions that have both high oxidative and physical stability^63^. The oxidative stability of both WPI and sodium caseinate for the stabilization of linoleic acid emulsions was investigated^64^. Caseins and other proteins have exhibited antioxidative features^65^.

Spreadable C oxidized over storage time, reaching around 9 meq O_2_/kg after 60 days of storage. Carrot juice is a rich source of carotenoids and phenolic compounds^66^, which retard oil oxidation. In the same context, pumpkin peel extract has the highest content of carotenoids, and phenol was used to prevent canola oil oxidation^67^. In addition, spreadable D (8.1 meqO_2_/kg) showed moderate oxidative stability due to its richness in polyphenolic compounds in doum^68,69^. It was noted that the oxidation of spreadable oleogels could be affected by their composition. Interestingly, these peroxide value patterns correlated with the hardness of the oleogels. That is, the harder the spreadable P became, the lower the peroxide value it had. This suggested that the restriction of oil mobility and migration via organogelation was effective in retarding oil oxidation during storage^6^.

##### Antioxidant activity

**Figure 5:**
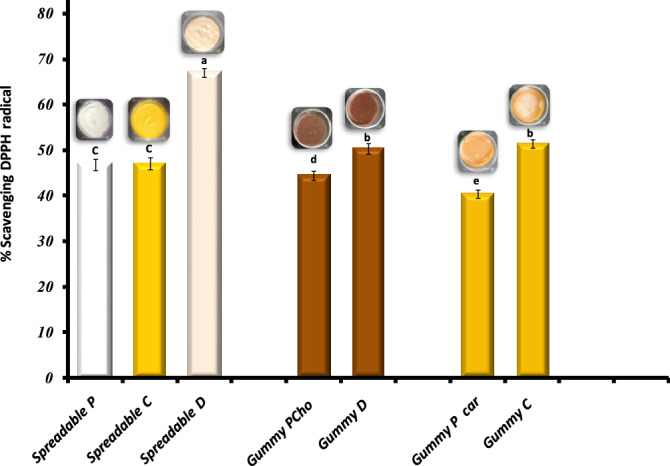
Antioxidant activity of functional spreadable (P, C, D) oleogel and nutritional gummies (P_cho_, D, P_car_, C,). Columns with similar letters are not statistically different according to DMRT (p ≤ 0.05).

Antioxidants play an important and vital role in retard oil oxidation as well as many health and biological benefits, so estimating their effects in food products is important^70,71^. Figure 5 shows the antioxidant activity of functional spreadable (P, D, and C) oleogels and nutritional gummies (P_cho_, D, P_car,_ and C). The spreadable D showed the best antioxidant activity (66.98%), which may be due to the high antioxidant content of doum extract and its juice that are combined in the same spreadable. The hot water extract from doum fruit palm is a rich and strong source of antioxidants. Also, spreadable P and C showed good antioxidant activity^72^. It was noted that a non-significant difference was found between spreadable P (46.83%) and C (47.05%). The same observation was made for gummy D and C. The antioxidant activity of gummy D (50.37%) and C (51.44%) was higher than gummy P_cho_ (44.40%) and P_car_ (40.37%) due to the presence of doum and carrot juice, respectively.

Many studies have shown the importance and role of beta-carotene that is present in carrot juice as a powerful antioxidant and has various therapeutic benefits, and it turns into vitamin A in the human body^73,74^. In addition, orange oil, which was also used in this study, has an antioxidant effect and is considered a natural and attractive flavor^75^. Milk proteins have antioxidant activity in scavenging reactive oxygen species^19^. As presented previously, the different spreadable oleogels and gummies in this study are considered excellent sources of antioxidants. Finally, all the selected materials used in this study were selected to play a functional role in the final produced product.

#### Effect of storage conditions

The effect of storage conditions at room temperature (30 °C) and refrigerator (4 °C) for 12 weeks on chemical composition (“Chemical composition”), color, pH, and oil binding capacity (OBC) for nutritional gummy formulations was studied. However, spreadable oleogel samples were kept only at 4 °C for 60 days.

##### Color stability

Table 4 shows the results regarding the color analysis of spreadable oleogel stored in a refrigerator for 60 days. Spreadable P showed the highest level of luminosity (L* value) compared to the other spreadable (O, D, and C) oleogels. It might be due to no color addition and the white color of spreadable ingredients (starch, maltodextrin, and casein). Positive b* values indicate a yellowish color, so spreadable C is much more yellow than spreadable D, P, and O oleogels due to the color of carrot juice^66^. The h* value refers to the hue’s location in the CIE-L*C*h color range. It was noted that the same trend was observed after the storage period except that spreadable O showed the highest h* and ΔE values. Both gummy P_cho_ and P_car_ slightly darkened (C* increased) in the refrigerator but in r.t. decreased. It was noted that h* was decreased in all prepared gummies in both storage times. Also, the color change in the gummy stored at room temperature and in the refrigerator for 12 weeks is detected in Table 4. Both gummy D and C showed the highest ΔE representing the highest amount of color change in the samples. While gummy P_cho_ and P_car_ displayed the lowest ΔE representing better protection of the oleogel at the initial time. After storage, the change in color in gummy D and C at r.t. is greater than the change in the refrigerator. It is very interesting to obtain results in the same context as the results of pH and oil binding capacity.

**Table 4:** Color characteristics of functional spreadable oleogels and gummy formulations stored at the initial time and after storage conditions (4 °C and r.t.).

Formulations	Refrigerator (4 °C)					
	L*	a*	b*	c*	h*	ΔE*
**Initial time**						
Spreadable O	33.02 ± 1.16a	1.18 ± 0.26a	4.85 ± 0.55a	4.85 ± 0.75a	84.20 ± 1.40a	0.69 ± 0.014b
Spreadable P	88.23 ± 0.76a	0.25 ± 0.05a	8.92 ± 0.29a	9.85 ± 0.45a	97.15 ± 5.35a	1.70 ± 0.021a
Spreadable D	72.40 ± 0.20a	4.90 ± 0.30a	21.17 ± 0.59a	21.97 ± 1.16a	76.70 ± 1.70a	0.26 ± 0.007a
Spreadable C	72.65 ± 0.15a	12.40 ± 0.30a	60.89 ± 2.11a	62.33 ± 1.65a	80.10 ± 1.40a	1.76 ± 0.042a
Gummy D	28.63 ± 0.06a	9.65 ± 0.25a	5.55 ± 0.05a	10.20 ± 0.70a	29.80 ± 1.30a	0.93 ± 0.014a
Gummy P_Cho_	33.53 ± 0.35a	11.53 ± 0.35a	7.90 ± 0.20a	14.15 ± 0.25a	34.50 ± 0.10a	0.17 ± 0.009b
Gummy C	43.63 ± 0.15a	10.75 ± 0.25a	24.10 ± 0.40a	25.25 ± 0.55a	67.85 ± 1.05a	1.42 ± 0.035a
Gummy P_Car_	49.20 ± 0.10a	10.30 ± 0.20a	23.75 ± 0.05a	25.55 ± 0.15a	67.10 ± 0.20a	0.41 ± 0.021a
r.t						
Gummy D	29.73 ± 0.25a	9.63 ± 0.06a	6.15 ± 0.15a	11.25 ± 0.25a	33.00 ± 0.20a	0.19 ± 0.014b
Gummy P_Cho_	31.93 ± 0.06a	11.30 ± 0.70a	7.40 ± 0.10a	13.05 ± 0.25a	34.52 ± 0.03a	0.54 ± 0.007b
Gummy C	45.93 ± 0.06a	10.65 ± 0.05b	25.50 ± 0.20b	27.70 ± 0.50b	68.45 ± 0.25a	0.54 ± 0.014b
Gummy P_Car_	47.37 ± 0.55a	9.83 ± 0.46a	22.75 ± 0.45a	24.60 ± 1.00a	67.00 ± 0.30a	0.24 ± 0.007b
**After storage**						
Spreadable O	34.03 ± 0.67a	1.25 ± 0.05a	0.80 ± 0.50b	1.50 ± 0.10b	179.45 ± 0.35b	2.86 ± 0.035a
Spreadable P	86.82 ± 0.80a	0.35 ± 0.25a	7.90 ± 0.60a	6.25 ± 0.65b	94.37 ± 1.04a	1.86 ± 0.028a
Spreadable D	65.70 ± 0.90b	4.80 ± 0.70a	20.65 ± 0.65a	22.60 ± 1.30a	75.90 ± 0.90a	1.45 ± 0.021b
Spreadable C	72.40 ± 0.50a	9.50 ± 0.40b	60.50 ± 4.10a	62.20 ± 1.30a	81.37 ± 1.16a	1.01 ± 0.028b
Gummy D	27.15 ± 0.05b	5.45 ± 0.05b	2.65 ± 0.05b	5.90 ± 0.40b	23.73 ± 1.10b	0.27 ± 0.014b
Gummy P_Cho_	27.10 ± 0.60b	12.50 ± 0.30b	8.05 ± 0.25a	15.40 ± 0.90a	33.25 ± 1.05a	0.54 ± 0.021a
Gummy C	36.75 ± 0.05b	7.47 ± 0.31b	14.05 ± 0.15b	15.65 ± 0.05b	62.43 ± 0.40b	0.28 ± 0.028b
Gummy P_Car_	47.85 ± 0.25b	10.35 ± 0.15a	23.30 ± 0.30a	25.80 ± 1.20a	66.75 ± 0.25a	0.43 ± 0.014a
r.t						
Gummy D	22.45 ± 0.05b	8.75 ± 0.15b	4.10 ± 0.10b	10.85 ± 0.35a	27.35 ± 0.35b	1.25 ± 0.021a
Gummy P_Cho_	26.93 ± 0.03b	6.35 ± 0.35b	3.35 ± 0.55b	6.35 ± 0.25b	28.15 ± 0.55b	0.83 ± 0.042a
Gummy C	39.72 ± 0.08b	17.00 ± 0.30a	29.10 ± 0.20a	32.05 ± 0.05a	60.75 ± 0.05b	1.75 ± 0.021a
Gummy P_Car_	42.10 ± 0.10b	9.23 ± 0.12a	19.20 ± 0.10b	20.35 ± 0.15b	64.75 ± 0.05b	0.96 ± 0.014a

Columns with similar letters are not statistically different according to DMRT (p ≤ 0.05). Statistical analysis was performed to distingue the significance at the initial time and after storage.

##### pH

Table 5 refers to the pH values and oil binding capacity of functional different spreadable oleogels and healthy nutritional gummy formulations at the initial time and after storage. The pH value of spreadable O was 4.05 at the initial time and this value slightly increased after storage to 4.12. At the initial time, the highest pH value was noted with spreadable C, followed by spreadable D, while the lowest pH was with spreadable P. After cold storage, the pH values increased and the samples were in the same line at 0 times. In the case of different gummy formulations at cold storage and initial time, gummy D was the highest gummy sample; gummy P_Cho_ was followed by gummy C and gummy P_Car_. After storage, gummy P_Cho_ was the highest, followed by gummy D, gummy P_Car_, and gummy C. While stored at room temperature, pH values were the following: gummy P_Cho_ was above them in values, followed by gummy D, gummy P_Car_, and at least gummy C, and after storage pH values were increasing in storage and it was in the same trend. Due to the difference in the pH values in addition to adding citric acid in gummy samples made by caramel and adding orange oil as well, which makes the pH go towards increased acidity and thus the decrease in pH value. The pH values of the different gummies agreed with those reported by^57^, who found the pH values ranged from 2.8 to 3.1. Doum, carrot, and gelatin in the case of gummies had an impact on protein content (see Fig. 2) by increasing the level of either the gummy or the spreadable, which means that by increasing the protein content, amino acids increase, affecting the pH of the product^76^.

**Table 5:** The oil binding capacity (OBC) and pH of the functional spreadable oleogels and healthy nutritional gummy formulations.

Formulations	Initial time		After storage	
	pH	OBC (%)	pH	OBC (%)
**Refrigerator**				
Spreadable O	4.05 ± 0.01	80.90 ± 0.004	4.12 ± 0.02	74.51 ± 0.32
Spreadable P	4.49 ± 0.007	94.28 ± 0.02	4.54 ± 0.04	83.41 ± 0.33
Spreadable D	4.96 ± 0.01	97.75 ± 0.04	5.11 ± 0.06	89.12 ± 2.05
Spreadable C	5.55 ± 0.007	97.89 ± 0.02	5.64 ± 0.03	89.43 ± 1.22
Gummy D	5.34 ± 0.007	99.89 ± 0.06	5.26 ± 0.07	99.77 ± 0.02
Gummy P_Cho_	5.01 ± 0.02	99.86 ± 0.06	5.53 ± 0.04	99.85 ± 0.07
Gummy C	3.01 ± 0.01	99.81 ± 0.06	3.07 ± 0.05	99.67 ± 0.02
Gummy P_Car_	3.025 ± 0.007	99.91 ± 0.06	3.13 ± 0.04	99.86 ± 0.00
r.t.				
Gummy D	5.05 ± 0.01	99.85 ± 0.07	5.34 ± 0.10	99.32 ± 0.08
Gummy P_Cho_	5.35 ± 0.007	99.79 ± 0.003	5.56 ± 0.03	99.33 ± 0.1
Gummy C	2.79 ± 0.007	99.82 ± 0.07	3.04 ± 0.11	99.28 ± 0.13
Gummy P_Car_	2.96 ± 0.02	99.80 ± 0.01	2.88 ± 0.07	99.73 ± 0.04

Values represent the means ± SD.

##### Oil binding capacity (OBC)

Oil binding capacity, also presented in Table 5, is defined as the grams of bound oil divided by the grams of solid fat. It is an important test that affects the acceptance and appearance of the product^77^. At the initial time, the OBC was the least in spreadable O and was followed by the spreadable P, D, and C. OBC was in all cases higher than 90%, with the only exception of spreadable O (80.90%). After storage, functional spreadable oleogel showed a reduction of OBC of about 6–10%, but nutritional gummy formulations revealed a slight change. Gummy P_Car_ and P_Cho_ were the highest values, followed by gummy D and C. Amongst spreadable, the OBC was in the order: spreadable O < spreadable P < spreadable D < spreadable C, which reflects the effect of composition on the capacity of oil binding. As for the storage at room temperature, the values were lower compared to storage at the temperature of the refrigerator. The results of oil binding capacity agreed with that detected by^78^, who prepared two types of oleogel carnauba wax with canola oil or beeswax with grapeseed oil at different concentrations. The high values of oil binding capacity in the spreadable and gummy samples compared to the spreadable O were due to the presence of milk proteins (10% casein and 10% whey protein) in the samples, in addition to gelatin in gummies. As well, the high ability to bind fat is also due to the presence of milk proteins, which have a high ability to bind fat because they contain binding sites and a high ability to emulsify^79,80^. As mentioned by^81^, the ability to retain oil in a structured network is a complex concept involving morphology, distribution, surface absorption, and surface roughness of particles in the system. The previously described results highlight that gelators forming crystals with a needle-like morphology resulted in a network with a more efficient entrapping oil capacity. The results are in the same context as the macroscopic feature of primary importance to the use of oleogel in the food system.

#### FTIR and morphological observations

##### FTIR-ATR

**Figure 6:**
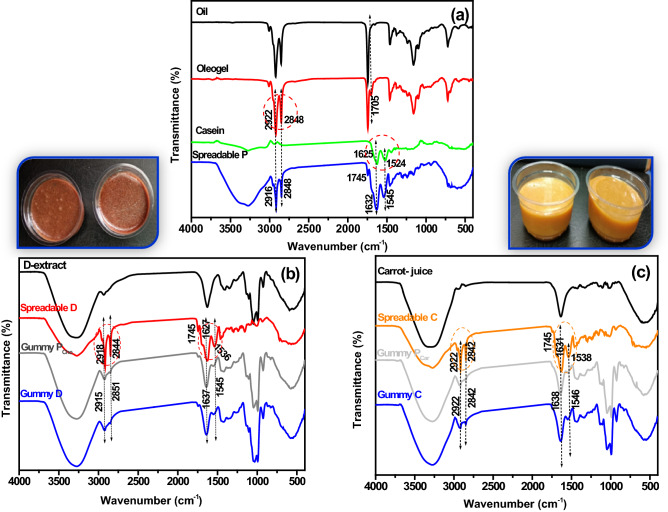
FTIR-ATR spectra of (**a**) spreadable P, (**b**) spreadable C, Gummy C, and (**c**) spreadable D, Gummy D.

To understand the possible changes in chemical functional groups in prepared formulations, FTIR-ATR spectra of canola oil, casein, carrot juice, doum juice, and gummy P, D, and C are demonstrated in Fig. 6^82^. The most prominent absorption band of canola oil at 1740 cm^−1^ can be assigned to the C=O stretching of aliphatic esters. The strong bands at around 2923 and 2855 cm^−1^ belong to the asymmetrical and symmetrical C–H stretching vibrations of CH_2_ groups. The band at around 1159 cm^−1^ may be assigned to the stretching of the C–O bonds of aliphatic esters or CH_2_ bending vibrations^83^. For oleogel formation, the peaks at 2916, 2855, and 1740 cm^−1^ can be observed for canola oil. The characteristic peak of stearic acid at 1705 cm^−1^ due to the stretching vibration of the carbonyl group was retained in the FTIR spectra of the oleogel. This may be due to the involvement of the carboxylic group of stearic acid in the noncovalent interactions (hydrogen bonding) during gelation^10^.

For spreadable formation, casein shows three absorption peaks at 1625 cm^−1^ (Amide I), 1531 cm^−1^ (Amide II), and 1235 cm^−1^ (Amide III), which can be attributed to the polypeptide backbone, the –COO– asymmetric stretching vibration of Asp and Glu, and the ring stretching vibration of the Tyr residues, respectively^84^. FTIR analysis of all spreadable revealed that the peaks at 1627–1632 cm^−1^ and 1536–1545 cm^−1^ widened and lengthened. However, the peaks at 2916–2922 cm^−1^, 2842–2848 cm^−1^, and 1747 cm^−1^ show lower peak intensity. This can be due to possible hydrogen bonding between the oleogel and casein, making the trapping of oleogel more stable. These results are acceptable with OBC results. Water molecules may be responsible for the peaks at 3281 and 3278 cm^−1^.

FTIR spectra mainly show the vibrations arising from the major gummy components (water, sugar, and gelatin). Sugar-related bands are mainly observed in the spectral region between 750 and 1500 cm^−1^. Three major bands (1258, 1348, 1412 cm^−1^) were observed between the 1500 and 1200 cm^−1^ spectral range, which is known for being a mixed region influenced by bending modes of > CH_2_ and CH_3_ groups in proteins and CH bending vibrations of carbohydrates^85^. Gelatin is one of the major components of gummy candies. In the FTIR spectra, we observe spectral features arising from the presence of gelatin. The most significant band (Amide I) related to gelatin is observed in the 1700–1600 cm^−1^ spectral range^86^. It was observed that the FTIR spectra of Gummy D appeared to be similar to the gummy C spectra. Based on these analytical results, it was demonstrated that quite an appropriate production recipe was applied to obtain the most commercial-like gummy candies.

##### TEM and PLM observation

**Figure 7:**
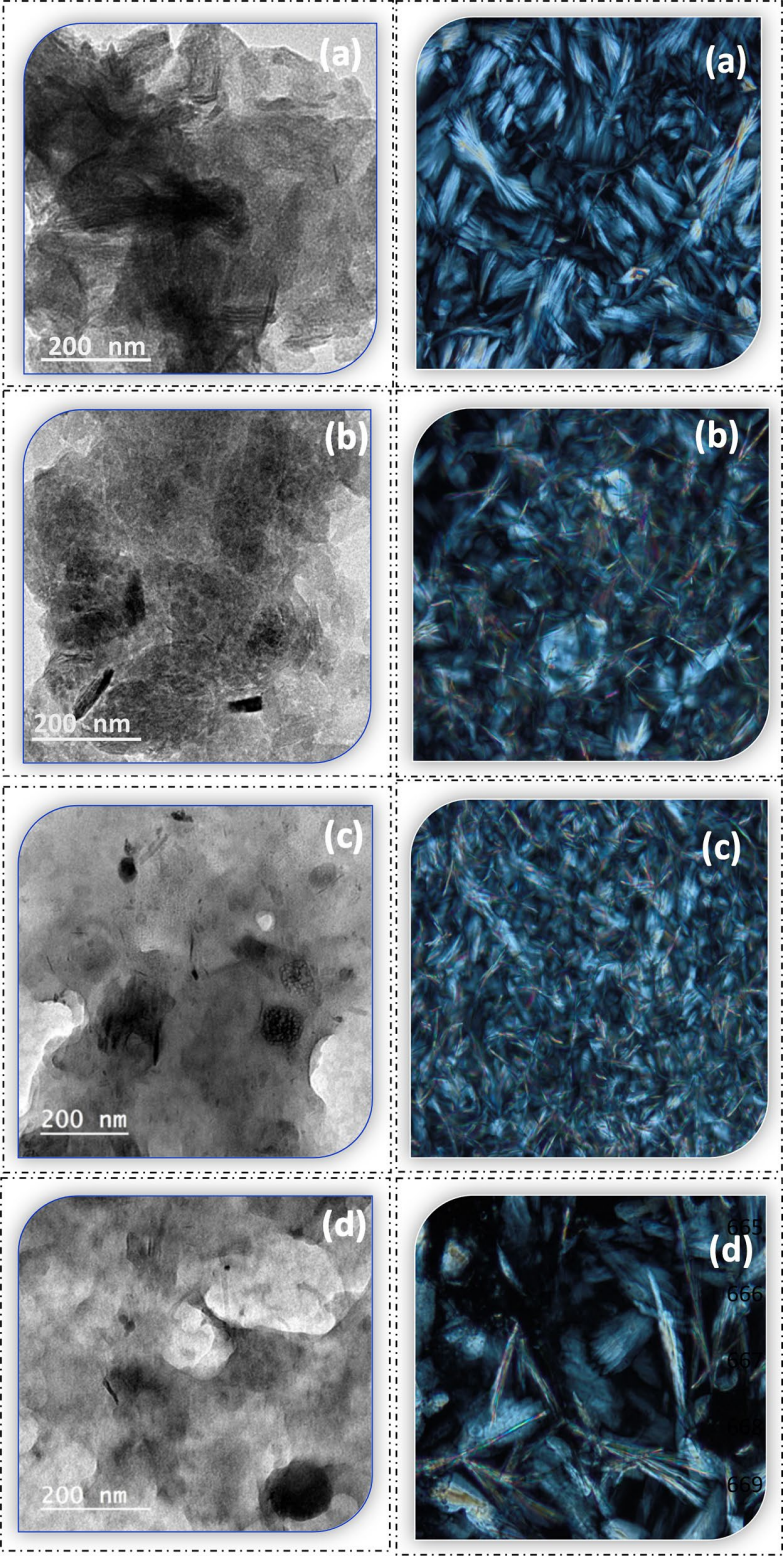
TEM (left) and PLM (right) micrographs of functional spreadable oleogel (**a**) spreadable O (**b**) spreadable P, (**c**) spreadable D, and (**d**) spreadable C.

**Figure 8:**
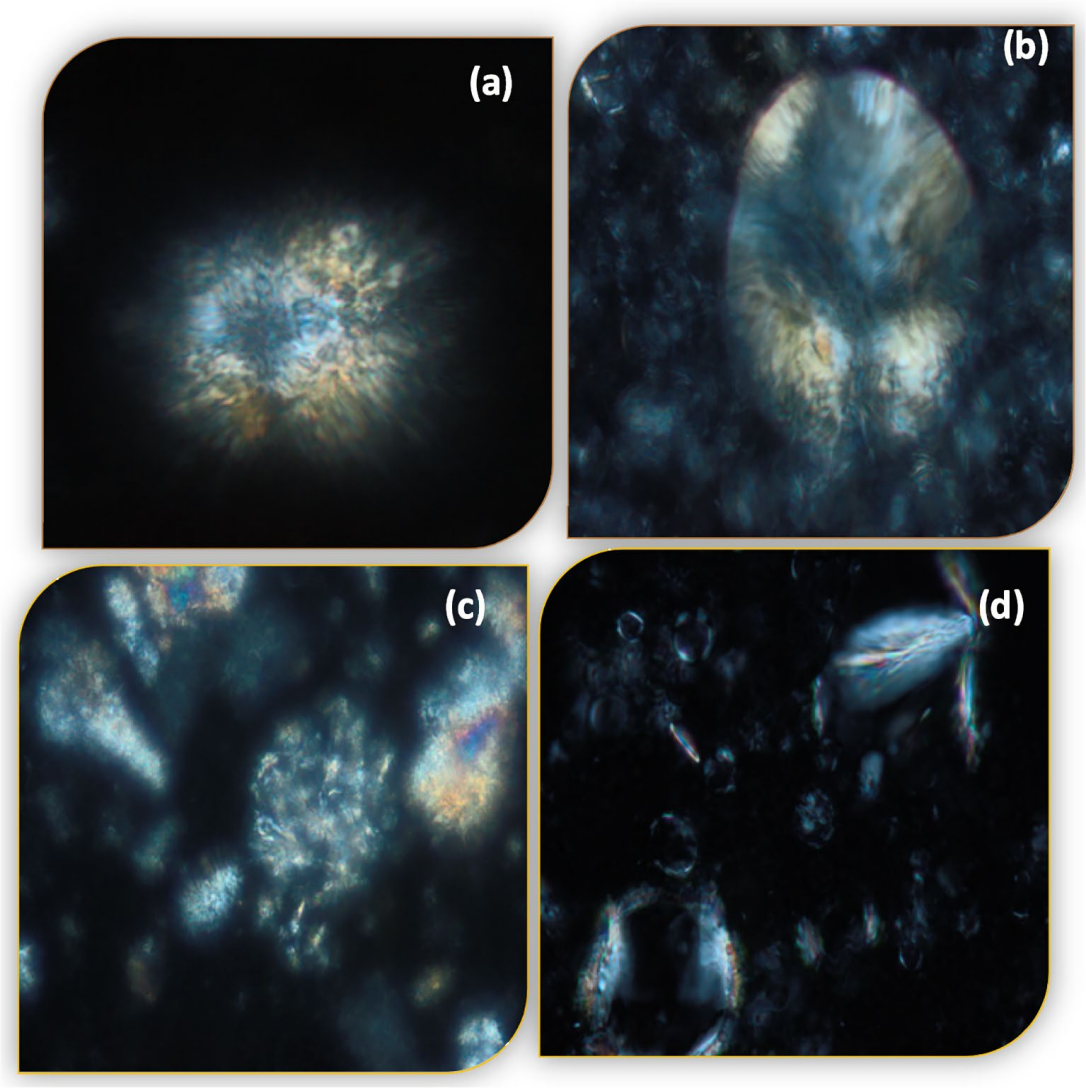
PLM of functional gummies based-spreadable oleogel (**a**) gummy P_Cho_ (**b**) gummy D, (**c**) gummy P_Car_, and (**d**) gummy C.

TEM and PLM observed the morphology and dispersion of spreadable O inside the functional spreadable oleogels and nutritional gummies. In the TEM micrographs, both spreadable O and P showed an aggregation network of oleogel needle shape. It was noted that spreadable D and C displayed a small needle of oleogel. This may be due to the aqueous phase of doum (D) and carrot (C) juice in the preparation process, which covered the needles of oleogel (Fig. 7). The PLM micrographs showed the presence of a coherent entangled network between oleogel and nutritional polymers. Spreadable P and D showed a small needle-like crystal morphology after gelling formation. A denser microstructure containing large needle-like crystals was observed in spreadable O and C, with larger aggregates in spreadable C that look like feathers (Fig. 7). Needle-like crystals were revealed in PLM microphotographs, similar to the results observed by^87^. Despite the opacity of the chocolate system, the needle crystals of functional spreadable oleogels were observed inside the chocolate and caramel gummies as stars at night (Fig. 8). A similar structure was also reported by^88^.

### Organoleptic test

Acceptance of dairy products is very important in food and is considered the main target, especially in this study. The additives used play an important role in the degree of acceptance of this food product type. The color and appearance of the product must be compatible with its taste. Consumers tend to choose foods with natural ingredients and natural colors because they are aware of the problems and damage of industrial materials. A famous saying is that a man eats with his eyes before his mouth. So, in this study, the authors concentrated on this target.

All the samples contained in this study are organoleptically accepted by all consumers. All samples tested, whether spreadable or gummy, in this study impressed all consumers of all age groups and cultural levels. The appearance was attractive, striking, manageable, and alterable, especially in the case of the gummies. The taste was delicious and attractive, as the panelists described it, especially the samples added to the orange oil and carrot juice, which were more attractive. The texture was known as the characteristics of the gumminess, but the spreads were individually viable and in line with the uses allocated to them. In general, these products, whether spreads or gummies, can be used as transport vessels for many important biocompatible compounds as well as drugs to be in an attractive form accepted by the consumer or patient as they are preferred for him. The results of the sensory arbitration correspond to those obtained by^37^ when he made gelatin candy using pineapple juice and carrot juice by 70:30, respectively. Also, the study carried out by^89^ studied the possibility of using some vegetables such as carrots and fruits such as strawberries in gelatin candy, which confirmed its sensory acceptance by the arbitrators. In another study, there was good acceptance of this oily pecan dessert as a healthy dessert version, low in saturated fatty acids and a source of oleic acid^90^.

### Approximate cost

The projected cost of the product is included in that study. For the oleogel, the cost of raw materials for the package, which contains 12.5 g and is calculated according to daily needs, is about 2 Egyptian pounds. The 25 g of spreadable oleogel are sufficient for daily needs and cost about 4.5 Egyptian pounds. While gummy candy cost the piece weighing 20 g, which also suffices for the daily needs of about 3 Egyptian pounds. Generally, the cost ranges from 2 to about 5 Egyptian pounds, which is equivalent to 0.13–0.32 dollars ($).

## Conclusion

The development of spreadable oleogels with various compositions enhances their possibility of being used in several food products. The chemical analysis revealed that developed spreadable oleogels and gummies are a good source of nutritional protein and fat. The highest PV was recorded in spreadable C, followed by D, while spreadable P and O showed good protection for oil. The maximum antioxidant activity was noted in spreadable D, gummy D, and C. Concerning storage studies, there was a little difference in pH and color as compared to the initial time. As shown by FTIR spectra, hydrogen bonding between functional spreadable oleogels and gummies was increased. These products (spreadable or gelatin candy) have been shown to have high sensory acceptance among consumers of all categories. As a result, markets require the provision of these nutrients in the form of appealing and multi-use products, such as fillings for baked goods, biscuits, or as a surface layer to decorate cakes and candy. Finally, further investigations are needed to study the antimicrobial activity of such products.

The patent number 2021/1287 was requested by the Egyptian Patent Office at the Academy of Scientific Research and Technology (ASRT).

## Data Availability

The datasets used and/or analysed during the current study available from the corresponding author on reasonable request.
